# Macrophage Phenotype Alterations in Treated Periodontitis Patients Suggest a Pro‐Inflammatory Bias

**DOI:** 10.1111/jre.70098

**Published:** 2026-03-11

**Authors:** Mariane Cristina Sloniak, Josiane Betim de Assis, Luciana Saraiva, Cláudio Mendes Pannuti, Ana Paula Lepique, Cristina Cunha Villar

**Affiliations:** ^1^ Discipline of Periodontics, Department of Stomatology School of Dentistry, University of São Paulo São Paulo Brazil; ^2^ Department of Immunology Institute of Biomedical Science, University of São Paulo São Paulo Brazil

**Keywords:** inflammation, macrophages, periodontitis, phenotype

## Abstract

**Aim:**

Understanding macrophage polarization in periodontitis is critical for elucidating the underlying disease mechanisms. This exploratory study aimed to characterize phenotypic features of monocyte‐derived macrophages (MDMs) from treated periodontitis patients.

**Methods:**

Peripheral blood mononuclear cells were isolated from 30 individuals classified into three groups: periodontal healthy (H) and patients with treated stage III, grade B (PIII‐B) or grade C (PIII‐C) periodontitis. Monocytes were differentiated into macrophages and cultured under non‐polarizing (M0), M1‐polarizing (LPS + IFN‐γ), or M2‐polarizing (IL‐4 + IL‐13) conditions. MDM polarization was evaluated by immunophenotyping and cytokine secretion profiling.

**Results:**

Under M0 conditions, the percentage of M1 MDMs decreased in the H group but remained stable in both periodontitis groups, particularly in PIII‐C, which exhibited a higher M1/M2 ratio. Following M1 stimulation, no significant differences in M1/M2 ratios were observed among groups. However, H MDMs increased VEGF and IL‐10 secretion compared with those from PIII‐B. Under M2 stimulation, PIII‐C MDMs exhibited a higher proportion of M1 cells and increased IL‐6 secretion, while PIII‐B MDMs demonstrated an elevated M1/M2 ratio and lower IL‐10 response, suggesting a shift toward a more pro‐inflammatory profile.

**Conclusion:**

In this exploratory study, MDMs from individuals with successfully treated stage III, grade B or C periodontitis exhibited a tendency toward a M1‐like phenotype across different polarization conditions, indicating the possible persistence of a pro‐inflammatory immune trait that requires confirmation in longitudinal and mechanistic studies.

## Clinical Relevance

**Background:**

Macrophages present in periodontitis lesions predominantly display a pro‐inflammatory M1 phenotype, which plays a central role in periodontal tissue destruction. However, it remains uncertain whether this phenotype is driven exclusively by local environmental stimuli or whether it may also reflect systemic alterations in circulating monocytes in individuals susceptible to periodontitis. To explore this question, we investigated the phenotypic profile of monocyte‐derived macrophages (MDMs) from individuals with a history of successfully treated periodontitis.

**Added Value of This Study:**

In this exploratory analysis, MDMs from successfully treated periodontitis patients exhibited a tendency toward an M1‐like phenotype. This finding suggests the presence of systemic immune traits associated with a pro‐inflammatory profile even after clinical resolution of the disease.

**Clinical Implications:**

Although exploratory, the observed tendency toward an M1‐like polarization in MDMs from treated periodontitis patients suggests that systemic immune characteristics may not fully parallel clinical resolution of disease. These findings highlight the need for longitudinal studies to determine whether such post‐treatment immune profiles have any relationship with periodontal stability or recurrence risk, and whether they could ultimately inform personalized monitoring and maintenance strategies in patients with a history of periodontitis.

## Graphical abstract

Monocyte‐derived macrophages from successfully treated stage III periodontitis patients show a persistent skewing toward an M1‐like pro‐inflammatory phenotype across polarization conditions, suggesting that immune dysregulation may persist despite clinical periodontal stability.

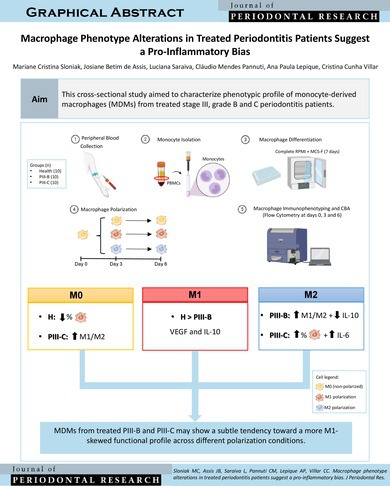

## 1 Introduction

Periodontitis results from interactions between dysbiotic biofilms and dysregulated immune responses, influenced by genetic, epigenetic, and environmental factors [1, 2, 3, 4]. Immune alterations observed in periodontitis may reflect pre‐existing host susceptibility, responses to dysbiotic biofilms, and/or adaptive changes driven by prior disease activity [5]; however, the relative contribution of these mechanisms remains unclear.

Host defense against periodontal infection involves coordinated innate and adaptive immune responses [6], with macrophages playing a crucial role in microbial clearance, regulation of inflammation, and tissue repair [7]. To perform these diverse functions, macrophages adopt a spectrum of activation states, classically simplified as pro‐inflammatory M1 phenotype, which facilitates pathogen clearance, and resolving M2 phenotype, which suppresses inflammation and promotes tissue healing [8, 9]. M1 predominance has been reported in periodontitis lesions, contributing to sustained inflammation and tissue destruction [10, 11, 12]. However, whether this skewed macrophage profile reflects only a local response to the periodontal microenvironment or broader host‐related immune traits remains unexplored.

In this exploratory study, we examined the phenotypic profile of peripheral blood monocyte‐derived macrophages (MDMs) from individuals with successfully treated periodontitis under regular supportive periodontal care (SPC), aiming to assess whether systemic immune features linked to susceptibility persist after disease clinical resolution. Given the exploratory design and the intentionally limited sample size, this study was conceived as hypothesis‐generating rather than a definitive assessment.

## 2 Methods

### 2.1 Study Design

This exploratory cross‐sectional study was conducted in the Discipline of Periodontics and in the Laboratory of Immunomodulation, Department of Immunology, at the University of São Paulo, Brazil. The study was conducted in accordance with the Declaration of Helsinki and Good Clinical Practice guidelines and was approved by the institutional ethics committee (Approval No. 1.981.738). All patients provided written informed consent before undergoing study‐related procedures.

### 2.2 Participants

Enrollment occurred between October 2019 and June 2021. Eligible subjects were aged 18–65, had good general health, and a minimum of 20 teeth (excluding third molars). They fell into three categories: (i) periodontal health (H), (ii) successfully treated stage III, grade B periodontitis (PIII‐B), and (iii) successfully treated stage III, grade C periodontitis (PIII‐C). H was defined as the absence of probing depths (PD) > 3 mm, < 10% of sites with bleeding on probing (BoP), and no attachment loss [13]. PIII‐B was characterized by a clinical attachment level (CAL) ≥ 5 mm in ≥ 30% of teeth and an age‐related bone loss index of 0.25–1.00 [1, 14]. PIII‐C was defined as CAL ≥ 5 mm in ≥ 30% of teeth, an age‐related bone loss index > 1.00, and familial aggregation [1, 14, 15, 16]. Patients with periodontitis were stratified into Grades B and C to capture differences in risk profile and susceptibility to disease progression. For this study, successful periodontal treatment was defined as < 10% of sites with BoP and no more than four sites with PD ≥ 5 mm [17] at the time of inclusion. All patients with periodontitis were enrolled while under regular SPC, ensuring a stable clinical condition and minimizing the potential influence of residual inflammation.

Exclusion criteria encompassed systemic conditions impacting periodontal disease progression, infectious diseases, use of immunosuppressants, bisphosphonates, or anti‐inflammatory drugs, pregnancy, nursing, and smoking.

### 2.3 Periodontal Treatment and Evaluation

Before enrollment, all periodontitis patients received full‐mouth subgingival instrumentation. PIII‐C (previously diagnosed as generalized aggressive periodontitis) additionally received adjunctive systemic antibiotics as part of their individualized treatment plan based on high disease risk and expected benefit, consisting of 400 mg metronidazole and 500 mg amoxicillin, three times daily for 14 days. After 45 days, clinical outcomes were reassessed and open flap debridement was performed when indicated. All patients then entered SPC, with follow‐ups every 3–6 months according to their risk profile.

At inclusion, a full‐mouth examination assessed PD, CAL, BoP, residual pockets (PD ≥ 5 mm), and the duration of SPC. All clinical parameters were measured at six sites per tooth (mesio‐buccal, mid‐buccal, disto‐buccal, mesio‐lingual, mid‐lingual, and disto‐lingual) on all teeth, excluding third molars, using a manual periodontal probe (PCP‐15, Hu‐Friedy).

### 2.4 Monocyte Isolation and Macrophage Differentiation

Sixty milliliters of peripheral blood were collected per participant. Peripheral blood mononuclear cells (PBMCs) were isolated using Ficoll‐Paque (PREMIUM 1073 g/mL, GE Healthcare, Uppland, Sweden) density gradient centrifugation. Then, PBMCs underwent negative magnetic cell separation (Human Pan Monocyte Isolation Kit, Miltenyi Biotec, NRW, Germany) for monocyte isolation. An anti‐CD14‐APC monoclonal antibody (clone M5E2; BD Biosciences, NJ, USA) confirmed a highly enriched monocyte population via flow cytometry (3.5‐fold enrichment). Isolated monocytes (2.6 × 10^6^ cells/ml) were resuspended in RPMI 1640 medium supplemented with 200 μM L‐glutamine, 100 IU/mL penicillin and 100 μg/mL streptomycin (Gibco, NE, USA). Next, the isolated cells were seeded onto 48‐well plates (Corning, NY, USA) and incubated at 37°C with 5% CO_2_ for 2 h for adhesion.

To induce macrophage differentiation, CD14^+^ adherent cells were cultured at 37°C, 5% CO_2_ in 500 μL differentiation medium [RPMI 1640 with 200 μM L‐glutamine, 10% FBS (fetal bovine serum) (Gibco, NE, USA), 100 IU/mL penicillin, 100 μg/mL streptomycin, and 20 ng/mL macrophage colony‐stimulating factor (M‐CSF) (PeproTech, NJ, USA)] [18] for 7 days. Medium was changed on the third day. The resulting population was named monocyte‐derived macrophages (MDMs). MDMs were differentiated using M‐CSF, which promotes the generation of a baseline population with an M2‐like bias, rather than granulocyte‐macrophage colony‐stimulating factor (GM‐CSF), which induces a more pro‐inflammatory (M1‐like) phenotype. This approach was chosen because macrophages in periodontitis lesions predominantly exhibit an M1‐like phenotype; therefore, the use of M‐CSF was considered more appropriate to avoid artificially amplifying pro‐inflammatory polarization.

### 2.5 Macrophage Polarization

**FIGURE 1:**
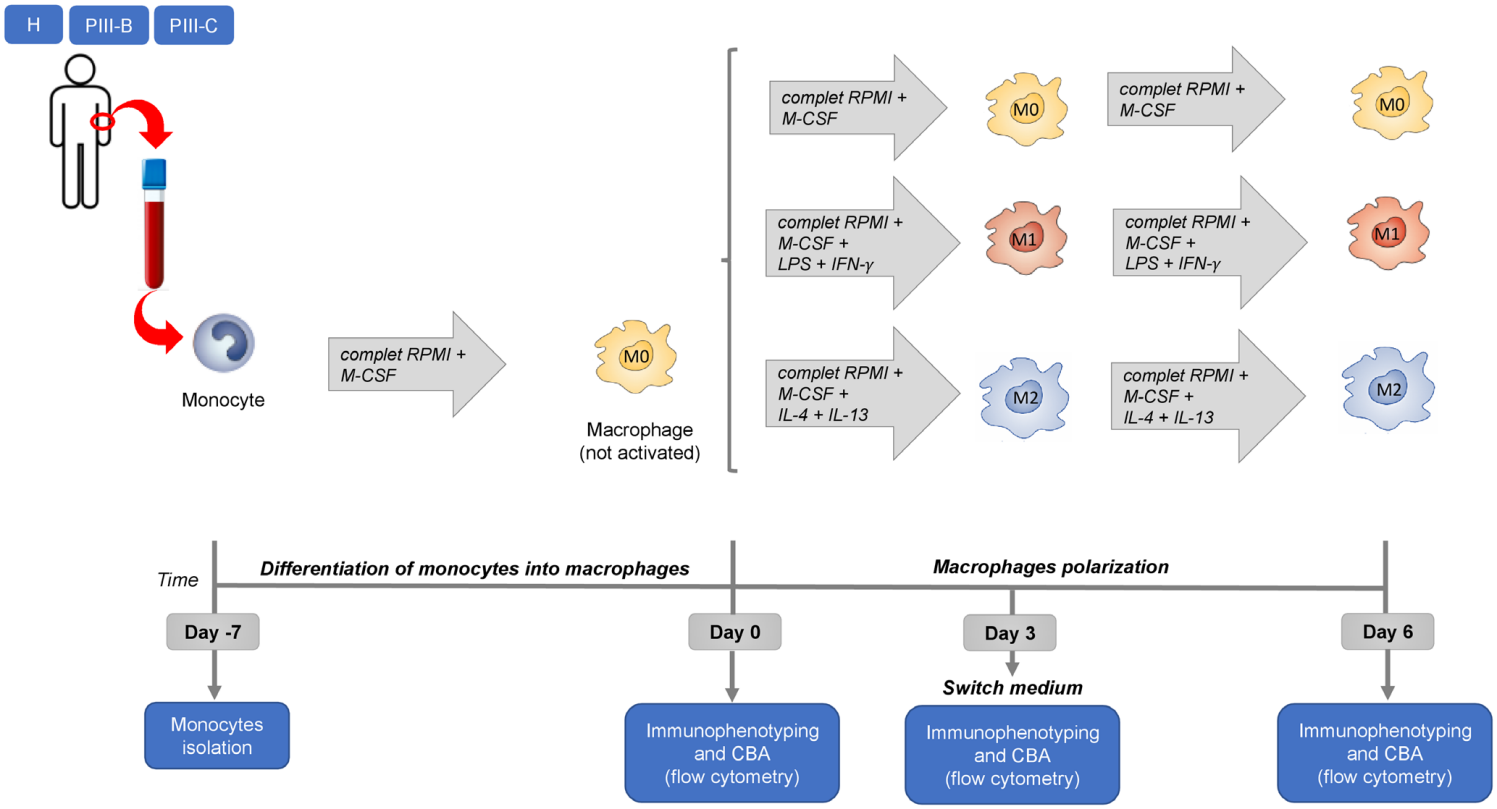
Study design. Peripheral blood samples from periodontal health (H), treated stage III, grade B periodontitis (PIII‐B), and treated stage III, grade C periodontitis (PIII‐C) groups were collected. CD14^+^ cells were isolated and cultured for 7 days in complete RPMI medium supplemented with M‐CSF for macrophage differentiation. Subsequently, monocyte‐derived macrophages underwent 6‐day phenotypic polarization stimuli, with day‐3 medium exchange (M0, M1, M2). Immunophenotyping and protein secretion analysis were performed on days 0 (baseline), 3, and 6 through flow cytometry.

For MDM polarization, the differentiation medium was replaced at day‐0 (D0) (baseline) with specific conditions, leading M0 (non‐polarized) MDMs to differentiate into both M1 (pro‐inflammatory) and M2 (pro‐healing) subpopulations (Figure 1). Protocols included:

- *M1*: differentiation medium + interferon‐γ (100 ng/mL) (PeproTech, NJ, USA) + lipopolysaccharide (LPS) from *Escherichia coli* O111:B4 (100 ng/mL) (Sigma‐Aldrich, MO, USA).
- *M2*: differentiation medium + interleukin (IL)‐4 (40 ng/mL) (PeproTech, NJ, USA) + IL‐13 (20 ng/mL) (PeproTech, NJ, USA).

MDMs in differentiation medium served as M0 controls. Conditions (M0, M1, M2) were maintained for 6 days, with media changed on day three.

### 2.6 Flow Cytometric Immunophenotyping

After Fc receptor blocking, 2 × 10^5^ cells were incubated with antibodies: anti‐HLA‐DR‐PE (clone LN3; Invitrogen‐Thermo Fisher, MA, USA), anti‐CD197‐APC (clone 3D12; Invitrogen‐Thermo Fisher, MA, USA), anti‐CD163‐PerCP‐Cy5.5 (clone GHI/61; BD Biosciences, NJ, USA), and anti‐CD206‐FITC (clone 15–2; Biolegend, CA, USA) for 20 min at 4°C in the dark. Then, cells were suspended in DAPI (4′,6′‐diamino‐2‐fenil‐indol) (Invitrogen‐Thermo Fisher, MA, USA) for viability assessment. Data were acquired on a Fluorescence‐Activated Cell Sorting (FACS) Canto II flow cytometer (BD Biosciences, NJ, USA) and analyzed using FlowJo v.10.0.7 (BD Biosciences, NJ, USA) software. M1 MDMs were defined as HLA‐DR and CD197 double‐positive cells (HLA‐DR^+^ CD197^+^ cells), while M2 MDMs were defined as CD163 and CD206 double‐positive cells (CD163^+^ CD206^+^ cells) (see Figure S1 for gating strategy). The M1/M2 ratio was determined by dividing the percentage of M1 MDMs by the percentage of M2 MDMs.

### 2.7 Cytokine Secretion Profile

Cell‐free culture supernatants were collected at days 0, 3, and 6 post‐differentiation to assess the functional phenotype of MDMs. The cytokine panel was selected to capture phenotypic and functional outputs of macrophage polarization rather than to investigate the upstream molecular mechanisms governing macrophage differentiation or activation, capturing key pro‐inflammatory (M1‐associated) and pro‐resolving or angiogenic (M2‐associated) outputs. TNF‐α (tumor necrosis factor alpha), IL‐6, IL‐8, CCL2/MCP‐1 (monocyte chemoattractant protein‐1), CCL5/RANTES, and CXCL9/MIG (monokine induced by gamma interferon) were included as markers of inflammatory activation, whereas IL‐10, TGF‐β (transforming growth factor‐β), and VEGF (vascular endothelial growth factor) were assessed as indicators of regulatory, pro‐resolving, and tissue‐repair–associated functions. Cytokine concentrations were measured using Cytometric Bead Arrays (BD Biosciences, NJ, USA). Data were acquired on a FACS Canto II flow cytometer and analyzed using FCAP Array software v.3.0.1 (BD Biosciences, NJ, USA). Protein levels were expressed in pg/mL.

### 2.8 Statistical Analyses

Statistical analyses were conducted using BioEstat 5.0 (BioEstat, Pará, Brazil) and Jamovi 2.7.6 (The Jamovi Project, Sydney, Australia) software. Given the exploratory, hypothesis‐generating nature of this study, the statistical approach, including sample size, covariate adjustment, and handling of multiplicity, was aligned with the primary objective of identifying putative biological signals rather than providing definitive effect estimates of associations. Accordingly, no a priori sample size calculation was performed. The cohort size was intentionally limited to enable phenotypic and functional immune profiling across multiple stimuli and time points.

Data normality was assessed using the Shapiro–Wilk test and homogeneity of variances was evaluated with the F test. For intergroup comparisons involving unpaired non‐parametric data, the Kruskal–Wallis test followed by Dunn's multiple comparisons test was employed. The effect of the covariate age in the intergroup analyses was evaluated using the ANCOVA test. Pairwise comparisons for repeated measures were performed using Friedman's test with Conover's post hoc analysis.

Given the limited sample size, the dataset was insufficient to support stable multivariable regression models with multiple covariates. Under these conditions, such models would be prone to overfitting, unstable parameter estimates, inflated standard errors, and reduced reproducibility, conveying a false sense of analytical rigor without providing reliable inference. For this reason, the analysis of the covariate's effect was restricted to age, which varied among groups. Regarding multiplicity, and consistent with the exploratory objective of the study and the high interrelatedness of the immunological variables, primary analyses were conducted using nominal *p*‐values without formal correction for multiple testing. Although strict correction would reduce the risk of type I error, it would substantially increase the risk of type II error in this small dataset and could obscure biologically meaningful signals relevant for hypothesis generation. A significance level of *p* < 0.05 was adopted for all tests.

## 3 Results

Ten patients were included in each group (Table 1). There were no significant differences among groups regarding sex distribution or duration of SPC. Although the PIII‐B group was older, age did not influence macrophage phenotype or cytokine secretion across baseline, M0, M1, and M2 conditions.

**TABLE 1:** Demographic characteristics and full‐mouth clinical periodontal parameters.

Variables	Groups		
	*H*	PIII‐B	PIII‐C
Sex (*n*)			
Male	7	6	6
Female	3	4	4
Age (years)			
Mean ± SD	30.1 ± 5.6	46.3 ± 8.0*	36.8 ± 5.3^#^
95% CI	(26.1–34.1)	(40.6–52.0)	(33.0–40.6)
Time since completion of active periodontal treatment (months)			
Mean ± SD	N/A	35.13 ± 5.25	37.63 ± 10.20
95% CI	N/A	(31.37–38.88)	(30.33–44.92)
Duration of supportive periodontal care (months)			
Mean ± SD	N/A	30.63 ± 5.93	34.50 ± 10.73
95% CI	N/A	(26.07–35.18)	(29.41–42.59)
Probing depth (mm)			
Mean ± SD	1.92 ± 0.56	2.71 ± 0.36*	2.73 ± 0.28*
95% CI	(1.56–2.27)	(2.51–2.92)	(2.59–2.87)
Bleeding on probing (%)			
Mean ± SD	7.01 ± 2.47	7.66 ± 1.74*	7.89 ± 1.27*
95% CI	(5.64–8.38)	(6.77–8.56)	(7.33–8.46)
Residual pockets ≥ 5 mm (*n*)			
Mean ± SD	0.00 ± 0.00	1.90 ± 0.57	2.10 ± 0.74
95% CI	(0.00–0.00)	(1.52–2.28)	(1.63–2.57)
Patient distribution according to the number of residual pockets ≥ 5 mm (*n*)			
0 sites	10	0	0
1 site	0	2	1
2 sites	0	7	8
3 sites	0	1	0
4 sites	0	0	1
Clinical attachment level (mm)			
Mean ± SD	1.52 ± 0.41	2.32 ± 0.79	3.58 ± 1.08 *^#^
95% CI	(1.2–1.84)	(1.79–2.86)	(2.98–4.18)

*Note:* Values are presented as mean ± SD (95% CI) or number (*n*). Statistical differences were estimated using the parametric ANOVA test, followed by Tukey's *post hoc* test (except for the variable *duration of supportive periodontal care—months*, which was analyzed using Student's *t*‐test, and sex distribution, which was evaluated using Pearson's Chi‐square test). Significant intergroup differences: * indicates *p* < 0.05 compared to H*; #* indicates *p* < 0.05 compared to PIII‐B. H = periodontal health; N/A = not applicable; PIII‐B = treated stage III, grade B periodontitis; PIII‐C = treated stage III, grade C periodontitis.

### 3.1 Baseline MDM Phenotypes Reveal Subtle Differences Between Grade B and Grade C Periodontitis

**FIGURE 2:**
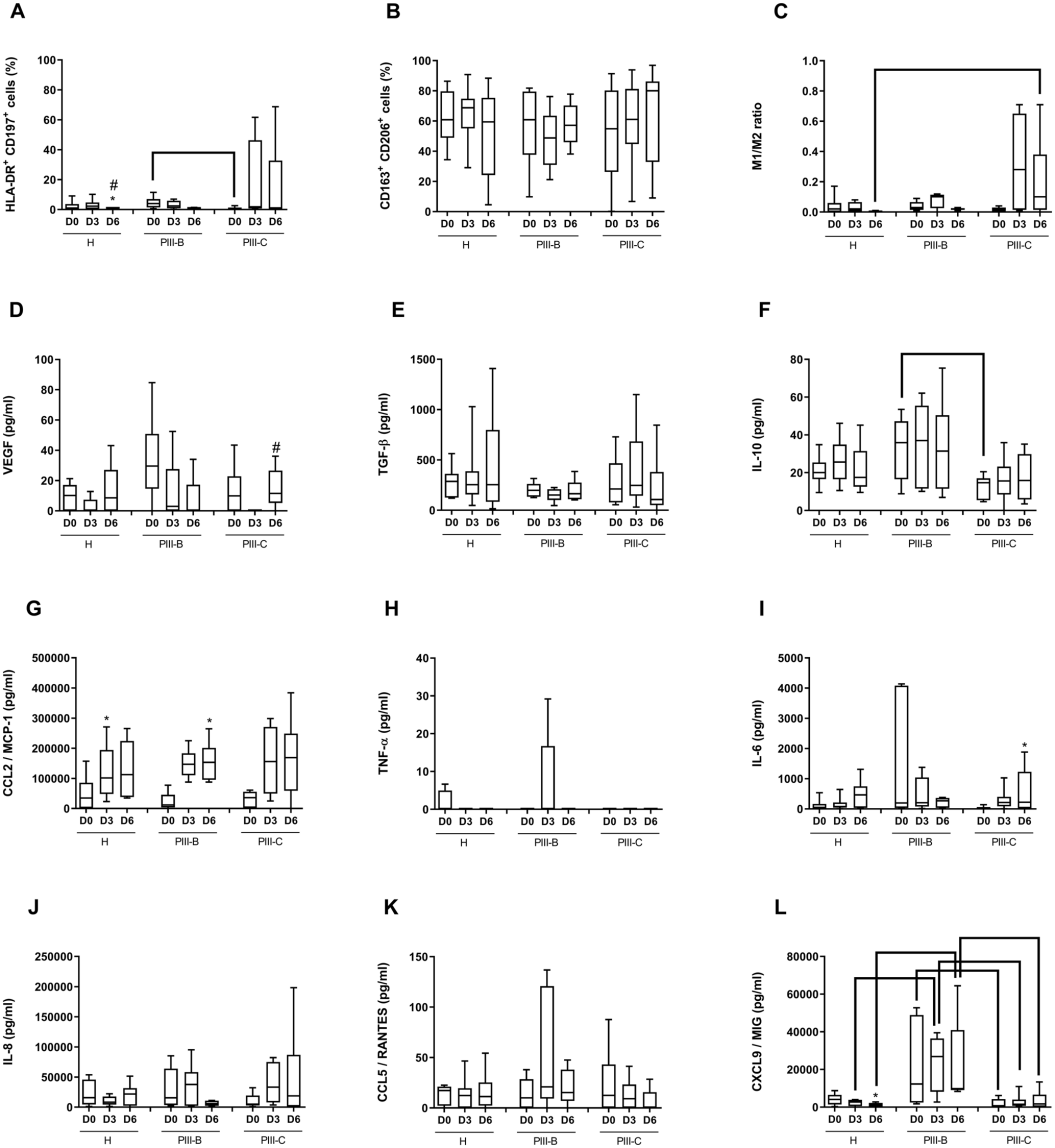
Monocyte‐derived macrophage phenotype at baseline (D0) and under M0 conditions. (A) Percentage of M1 (HLA‐DR^+^ CD197^+^) macrophages. (B) Percentage of M2 (CD163^+^ CD206^+^) macrophages. (C) M1/M2 ratio. (D–L) Protein secretion profile. Each box shows median values; box heights symbolize the IQR (Q3–Q1); Q3 and Q1 quartiles correspond to the top and bottom boundaries of the box, respectively; whiskers indicate values > Q3 (top: Max—Q3) or < Q1 (bottom: Q1—min). Intragroup differences were estimated using non‐parametric Friedman's test and Conover's post test; intergroup differences used non‐parametric Kruskal‐Wallis, followed by Dunn's *post hoc*. Significant intragroup differences: * indicates *p < 0.05* compared with D0*; #* indicates *p < 0.05* compared with D3. Black bars show intergroup differences (*p* < 0.05). D = day; H = periodontal health; IQR = interquartile range; PIII‐B = treated stage III, grade B periodontitis; PIII‐C = treated stage III, grade C periodontitis.

At day 0, MDMs exhibited a predominantly M2‐like phenotype. The proportions of M1 and M2 MDMs and the M1/M2 ratio did not differ between H and the periodontitis groups (Figure 2A–C). However, PIII‐B showed a higher percentage of M1 MDMs than PIII‐C (Figure 2A), with higher IL‐10 and CXCL9 secretion (Figure 2F,L). Additionally, a subset of individuals in the PIII‐B group exhibited elevated IL‐6 levels, resulting in a wider interquartile range (Figure 2I).

### 3.2 Under Non‐Polarizing Conditions, Periodontitis MDMs Display Selective Inflammatory Alterations

Under non‐polarizing (M0) conditions, M1 percentages decreased over time in the H group but remained stable in both PIII‐B and PIII‐C groups (Figure 2A). Notably, a subset of patients in the PIII‐C group demonstrated an increase in M1‐like cells (Figure 2A), resulting in a significantly higher M1/M2 ratio in the PIII‐C group compared with that of the H group by Day 6 (Figure 2C). In contrast, M2 percentages remained stable across all groups (Figure 2B). Over time, MDMs from the PIII‐C group exhibited fluctuations in VEGF secretion and a sustained increase in IL‐6 secretion (Figure 2D,I). While CXCL9 secretion decreased in the H group, it remained consistently elevated in the PIII‐B group under M0 conditions (Figure 2L).

### 3.3 M1 Polarization Induces Similar Pro‐Inflammatory Responses Across Groups With Limited Group‐Specific Variations

**FIGURE 3:**
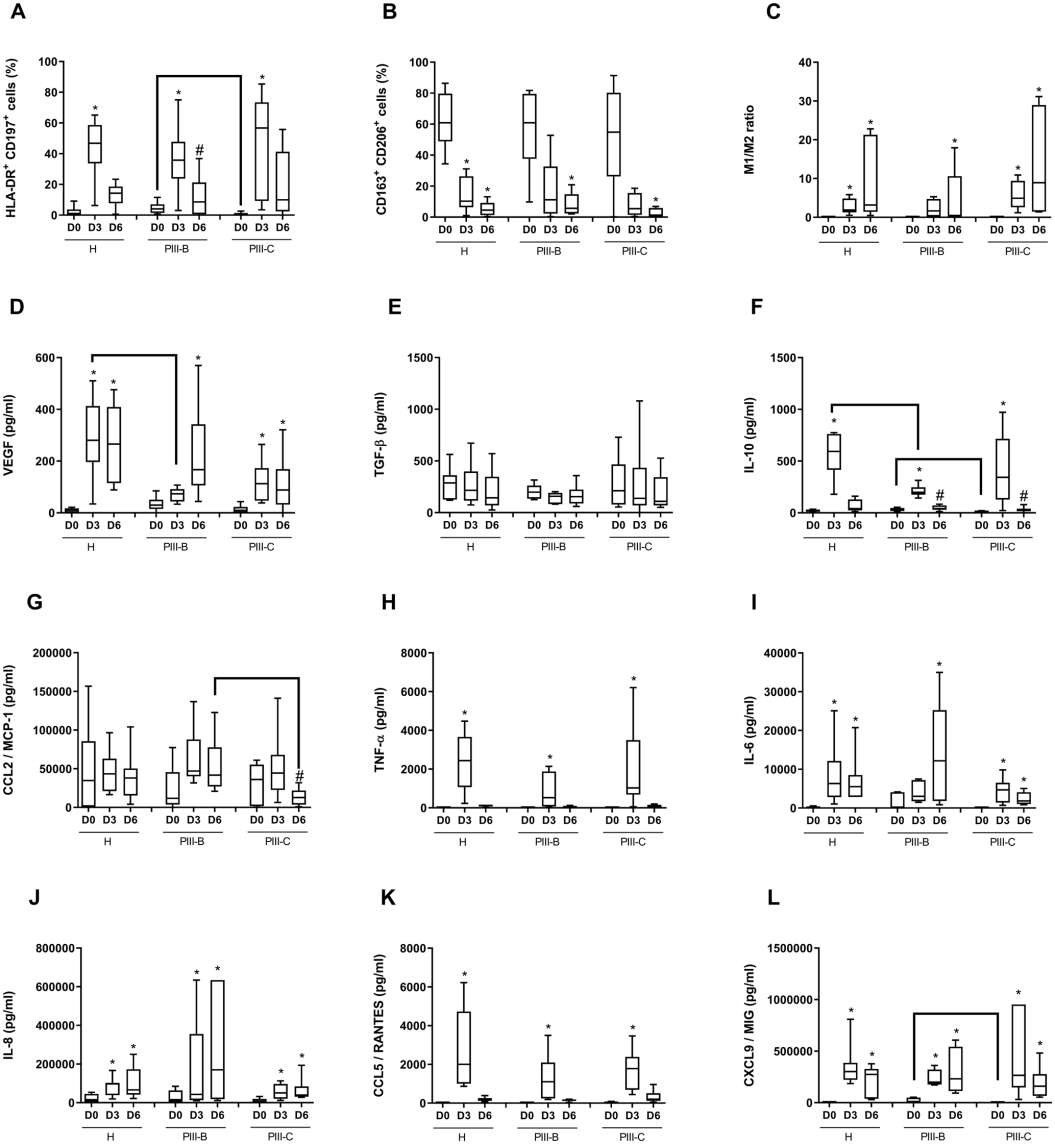
Monocyte‐derived macrophage phenotype at baseline (D0) and under M1 conditions. (A) Percentage of M1 (HLA‐DR^+^ CD197^+^) macrophages. (B) Percentage of M2 (CD163^+^ CD206^+^) macrophages. (C) M1/M2 ratio. (D‐L) Protein secretion profile. Each box shows median values; box heights symbolize the IQR (Q3–Q1); Q3 and Q1 quartiles correspond to the top and bottom boundaries of the box, respectively; whiskers indicate values > Q3 (top: Max—Q3) or < Q1 (bottom: Q1—min). Intragroup differences were estimated using non‐parametric Friedman's test and Conover's post test; intergroup differences used non‐parametric Kruskal‐Wallis, followed by Dunn's *post hoc*. Significant intragroup differences: * indicates *p < 0.05* compared with D0*; #* indicates *p < 0.05* compared with D3. Black bars show intergroup differences (*p < 0.05*). D = day; H = periodontal health; IQR = interquartile range; PIII‐B = treated stage III, grade B periodontitis; PIII‐C = treated stage III, grade C periodontitis.

Under M1 polarization conditions, all groups showed increased proportions of M1 MDMs and decreased proportions of M2 MDMs, resulting in changes in M1/M2 ratios; however, no significant differences were observed among groups (Figure 3A–3C). M1 percentages peaked at day 3 and declined by day 6, paralleled by IL‐10, TNF‐α, CCL5, and CXCL9 secretion (the latter only in the H and PIII‐C groups) (Figure 3F,H,K,L). This temporal pattern may reflect an attempted regulatory response rather than cell death, as cell viability remained stable (Figure S2). MDMs from the H group secreted higher levels of VEGF and IL‐10 compared with those of the PIII‐B group, suggesting a more efficient anti‐inflammatory regulation (Figure 3D,F); whereas PIII‐B secreted more CCL2 than PIII‐C (Figure 3G). Across all groups, IL‐6 and IL‐8 secretion increased over time (Figure 3I,J), while TGF‐β remained stable (Figure 3E).

### 3.4 M2 Polarization Elicits Distinct but Limited Inflammatory Responses in Periodontitis MDMs

**FIGURE 4:**
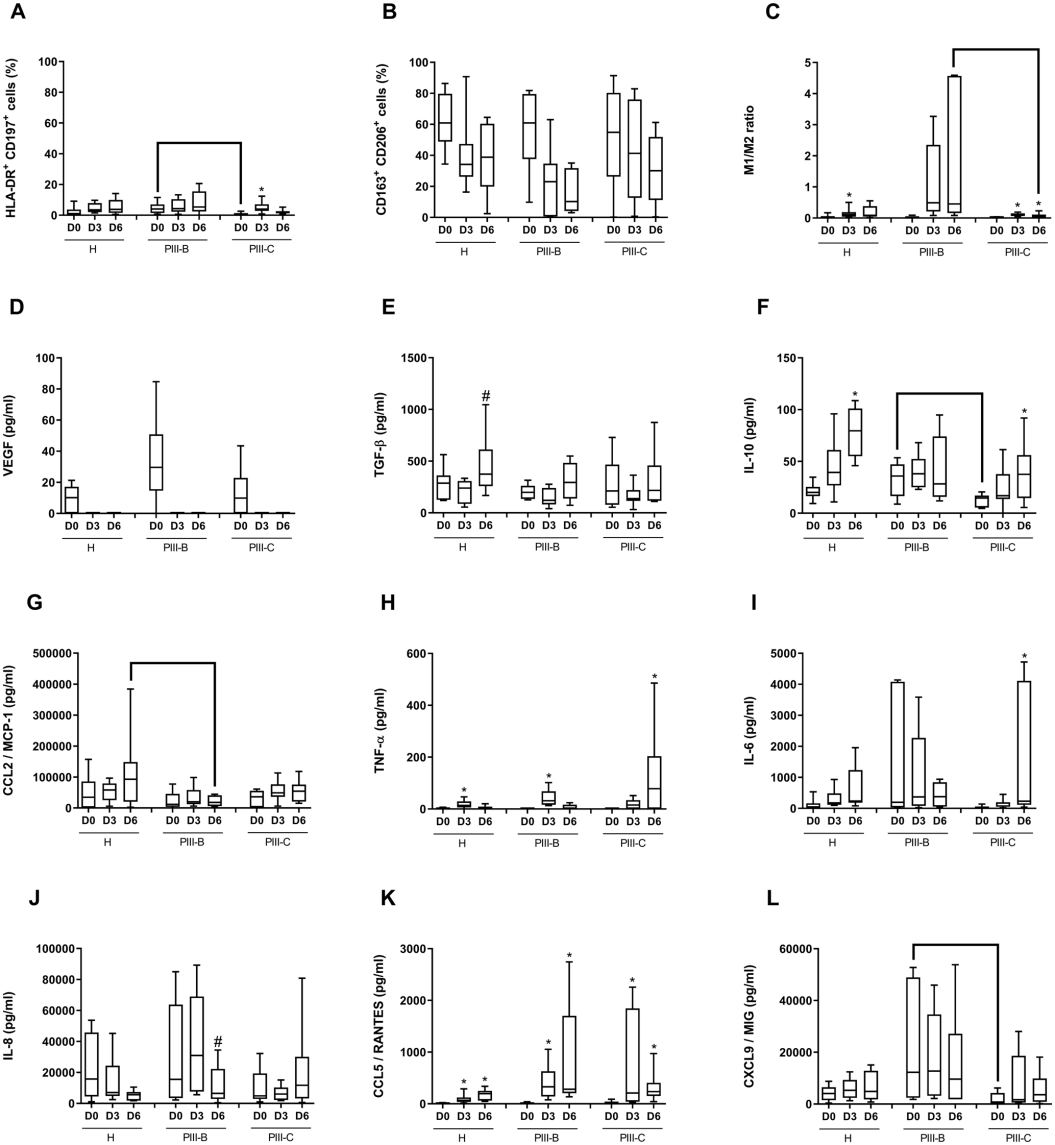
Monocyte‐derived macrophage phenotype at baseline (D0) and under M2 conditions. (A) Percentage of M1 (HLA‐DR^+^ CD197^+^) macrophages. (B) Percentage of M2 (CD163^+^ CD206^+^) macrophages. (C) M1/M2 ratio. (D‐L) Protein secretion profile. Each box shows median values; box heights symbolize the IQR (Q3–Q1); Q3 and Q1 quartiles correspond to the top and bottom boundaries of the box, respectively; whiskers indicate values > Q3 (top: Max—Q3) or < Q1 (bottom: Q1—min). Intragroup differences were estimated using non‐parametric Friedman's test and Conover's post‐test; intergroup differences used non‐parametric Kruskal‐Wallis, followed by Dunn's *post hoc*. Significant intragroup differences: * indicates *p* < 0.05 compared with D0*; #* indicates *p < 0.05* compared with D3. Black bars show intergroup differences (*p* < 0.05). D = day; H = periodontal health; IQR = interquartile range; PIII‐B = treated stage III, grade B periodontitis; PIII‐C = treated stage III, grade C periodontitis.

Under M2‐polarizing conditions, M1 percentages remained stable in the H and PIII‐B groups but significantly increased after 3 days in the PIII‐C group (Figure 4A). In contrast, M2 MDM percentages remained unchanged across all groups (Figure 4B). The M1/M2 ratio increased in both H and PIII‐C groups in response to M2 stimulation, whereas it remained stable in the PIII‐B group (Figure 4C). By Day 6, however, the M1/M2 ratio in the PIII‐B group exceeded that of the PIII‐C group, driven by a decrease in M2 percentages and an increase in M1 percentages in a subset of PIII‐B patients (Figure 4A,B).

Cytokine secretion profiles were also altered following M2 stimulation. TGF‐β levels increased in the H group (Figure 4E) and IL‐10 rose in both the H and PIII‐C groups (Figure 4F). The PIII‐B group exhibited a reduction in IL‐8 levels by day 6 (Figure 4J); whereas PIII‐C exhibited higher TNF‐α and IL‐6 levels (Figure 4H,I). Additionally, on day 6, MDMs from the H group secreted higher levels of CCL2 compared with those of the PIII‐B group (Figure 4G).

## 4 Discussion

As an exploratory study based on a deliberately small cohort, our findings should be interpreted as hypothesis‐generating rather than confirmatory. In this context, we observed that MDMs from individuals with successfully treated stage III periodontitis exhibit a subtle tendency toward an M1‐like profile across different polarization environments (M0, M1, and M2). These findings raise the possibility that certain inflammatory traits may persist after successful clinical therapy. This association could reflect several non–mutually exclusive scenarios, including pre‐existing host susceptibility, long‐lasting immune imprinting from prior disease activity, and/or treatment‐related immune modulation. Moreover, because successful periodontal treatment is defined solely by clinical parameters, it remains unclear whether complete immunological resolution had been achieved at the time of sampling; therefore, observed phenotypes may reflect delayed immunological normalization. Given the dynamic nature of macrophage polarization, these patterns could also represent transient activation states. However, given the cross‐sectional design, our results cannot determine directionality, persistence, or causality and therefore cannot distinguish among these possibilities.

At baseline, MDMs from both PIII‐B and PIII‐C patients exhibited phenotypes broadly comparable to those of healthy individuals, suggesting that differences are not constitutively present in circulating monocytes. Within the treated periodontitis cohort, MDMs from PIII‐B patients secreted higher levels of IL‐10 and CXCL9 than those from PIII‐C patients. CXCL9 promotes Th1 cell recruitment and osteoclast differentiation [19] and is typically elevated in untreated periodontitis [20]. Conversely, IL‐10 suppresses key pro‐inflammatory pathways implicated in periodontitis pathogenesis [21, 22]. Consistent with our findings, higher IL‐10 levels have been reported in moderate compared with rapidly progressing forms of periodontitis [23]. The simultaneous increase of IL‐10 and CXCL9 by PIII‐B MDMs likely reflects the coexistence of regulatory and inflammatory pathways, consistent with macrophage functional heterogeneity.

Under M0 conditions, MDMs from healthy individuals reduced M1‐like cells over time, whereas MDMs from treated periodontitis patients maintained more stable M1 proportions, with higher M1/M2 ratios and sustained IL‐6 secretion, particularly in PIII‐C. IL‐6, a cytokine closely associated with periodontitis [24], promotes neutrophil recruitment [25] and prolongs their survival by inhibiting apoptosis [26]. In addition, whereas CXCL9 secretion declined over time in healthy individuals, it remained elevated in the PIII‐B group. Together, these findings suggest that MDMs from treated periodontitis patients tend toward a more inflammatory profile under non‐polarizing conditions, which could increase the risk of disease recurrence [27].

Under M1‐polarizing conditions, all groups showed robust induction of the M1 phenotype and comparable M1/M2 ratios, indicating preserved pro‐inflammatory capacity in both health and treated periodontitis. The concurrent increase in VEGF and IL‐10 across groups in response to M1 polarizing conditions likely reflects compensatory regulatory mechanisms following strong inflammatory stimulation. Notably, MDMs from healthy individuals secreted higher levels of VEGF and IL‐10 than those from PIII‐B patients, suggesting greater ability to counterbalance inflammation. Although some mediators differed between PIII‐B and PIII‐C (e.g., CCL2), these contrasts were inconsistent across other conditions (M0 and M2). Nonetheless, higher CCL2 secretion by PIII‐B MDMs under M1 stimulation is consistent with reports of higher CCL2 levels in chronic than aggressive periodontitis [23, 28]. Given CCL2's role in promoting Th2 polarization [29] and preventing alveolar bone loss in periodontitis [30], its increased secretion by PIII‐B MDMs may be compatible with differences in immune regulatory networks associated with slower disease progression in these patients relative to those with PIII‐C, although this remains speculative.

Under M2‐polarizing conditions, M2 MDM percentages remained stable across all groups. However, MDMs from the PIII‐C group exhibited an increase in M1‐like cells accompanied by elevated TNF‐α and IL‐6 secretion, suggesting less complete skewing toward a pro‐resolving profile in response to stimuli typically associated with resolution of inflammation [31]. These findings are consistent with previous reports of increased IL‐6 production by monocytes from patients with aggressive periodontitis [32]. While M1 and M2 percentages did not differ significantly among groups under M2 stimuli, the elevated M1/M2 ratio and diminished IL‐10 response observed in PIII‐B MDMs further suggest a relative shift toward a more inflammatory functional profile.

Overall, differences between PIII‐B and PIII‐C MDMs were modest and inconsistent across polarization conditions, providing little support for clear grade‐specific immune signatures. Thus, the central finding of this study relates to the contrast between treated periodontitis and periodontal health, whereas grade‐related patterns should be viewed as exploratory. Importantly, the PIII‐C group had previously received adjunctive systemic antibiotics, a major potential confounder that limits comparability between groups. Although treatment was completed on average 35 months before inclusion, we cannot exclude lasting effects of prior antimicrobial exposure on innate immune programming. Consequently, any apparent immunological differences between Grades B and C should be interpreted with caution, as antibiotic exposure is a plausible alternative explanation.

Several aspects are important for interpreting our findings. By studying only successfully treated patients, we minimized the confounding influence of dysbiotic biofilms and ongoing inflammation on circulating cells [33], but this approach also precluded assessment of MDM behavior during active disease. Despite the diversity in macrophage ontogeny and function, our study focused exclusively on monocyte‐derived macrophages, as they are the predominant macrophage population in peripheral tissues during inflammation, infection, and injury. Lastly, macrophage polarization is highly plastic and dynamic, existing along a continuum rather than discrete M1/M2 states. Our conservative gating strategy, restricted to double‐positive M1 or M2 populations, enhanced specificity but likely excluded intermediate, mixed, and double‐negative phenotypes that may be biologically relevant.

Despite the insights generated, this study has several limitations that warrant consideration. As an exploratory, hypothesis‐generating investigation, no a priori sample size calculation was performed. Consequently, the relatively small cohort may have reduced the statistical power to detect subtle differences, with some observations reflecting trends rather than statistically significant effects. This approach, however, is consistent with early‐phase immunological and oncological studies, where the primary objective is hypothesis generation and the detection of putative biological signals [34, 35]. On the other hand, our study involved a large number of statistical comparisons across groups, time points, and polarization conditions, without correction for multiplicity. This approach was chosen to avoid an excessive increase in type II error in this small, highly exploratory dataset with correlated immunological variables, which could have obscured biologically meaningful signals relevant for hypothesis generation. Nevertheless, we acknowledge that the absence of formal correction for multiple testing increases the risk of type I error, and therefore our findings should be interpreted as preliminary plausible signals and require confirmation. It is also important to note that immune profiles may also vary considerably between patients long‐stable and those recently treated. Thus, future studies should include more heterogeneous populations followed longitudinally. Lastly, residual confounding could not be entirely excluded. Systemic inflammatory biomarkers such as serum C‐reactive protein (CRP) were not measured, and unrecognized systemic inflammation could have influenced MDM behavior. Similarly, prior antibiotic exposure in PIII‐C patients, as well as other unmeasured host or environmental factors, may have contributed to interindividual variability.

## 5 Conclusions

In conclusion, this exploratory study suggests a subtle tendency toward a more M1‐skewed profile in MDMs from patients with treated stage III periodontitis. However, given the cross‐sectional design and limited sample size, these findings are preliminary and hypothesis‐generating rather than definitive evidence of persistent systemic immune reprogramming.

## Author Contributions

**Mariane Cristina Sloniak:** substantial contributions to design of study, acquisition of data, analysis and interpretation of data, drafting the article, revising it critically for important intellectual content, final approval of the version to be published. **Josiane Betim de Assis:** acquisition of data, analysis and interpretation of data, drafting the article, revising it critically for important intellectual content, final approval of the version to be published. **Luciana Saraiva:** acquisition of data, final approval of the version to be published. **Cláudio Mendes Pannuti:** contributions to design of study, analysis and interpretation of data, drafting the article, revising it critically for important intellectual content, final approval of the version to be published. **Ana Paula Lepique:** substantial contributions to design of study, analysis and interpretation of data, drafting the article, revising it critically for important intellectual content, final approval of the version to be published. **Cristina Cunha Villar:** substantial contributions to conception and design of study, analysis and interpretation of data, drafting the article, revising it critically for important intellectual content, final approval of the version to be published. **Cristina Cunha Villar** and **Ana Paula Lepique** contributed equally as senior authors of this work.

## Funding

This study was supported by a research grant from the Osteology Foundation (Switzerland; grant # 16‐159) and a scholarship from the Coordenação de Aperfeiçoamento de Pessoal de Nível Superior (CAPES) (Brazil).

## Disclosure

*AI statement*: This manuscript used NLP (ChatGPT) for proofreading an entirely human‐generated text, but no other use of AI was made.

## Ethics Statement

This study protocol was approved by the institutional ethics committee (Approval No. 1.981.738).

## Consent

All participants provided written informed consent prior to inclusion in the study, in accordance with the Declaration of Helsinki and institutional guidelines.

## Conflicts of Interest

The authors declare no conflicts of interest.

## Supporting information


**Figure S1:** Representative macrophage gating strategy for flow cytometry (Days 0, 3 and 6). (A) Exclusion of debris, (B) selection of singlets, (C) determination of live cells (DAPI^−^), (D) M1 monocyte‐derived macrophages (percentage HLA‐DR^+^CD197^+^ cells) and (E) M2 monocyte‐derived macrophages (percentage CD163^+^CD206^+^ cells).
**Figure S2:** Cell viability during the polarization time course. (A) Percentage de macrophages at baseline (D0) and under M0 conditions. (B) Percentage de macrophages at baseline (D0) and under M1 conditions. (C) Percentage de macrophages at baseline (D0) and under M2 conditions. Each box shows median values; box heights symbolize the IQR (Q3–Q1); Q3 and Q1 quartiles correspond to the top and bottom boundaries of the box, respectively; whiskers indicate values > Q3 (top: max—Q3) or < Q1 (bottom: Q1—min). Intragroup differences were estimated using non‐parametric Friedman's test and Conover's post test; intergroup differences used non‐parametric Kruskal‐Wallis, followed by Dunn's *post hoc*. Significant intragroup differences: * indicates *p* < 0.05 compared with D0; Black bars show intergroup differences (*p* < 0.05). D = day; H = periodontal health; IQR = interquartile range; PIII‐B = treated stage III, grade B periodontitis; PIII‐C = treated stage III, grade C periodontitis.

## Data Availability

The datasets generated and analyzed during the current study are available from the corresponding author upon request.
