# Supplementary material for: Macrophage Phenotype Alterations in Treated Periodontitis Patients Suggest a Pro‐Inflammatory Bias
Source: J Periodontal Res. 2026 Mar 11;61(6):705–15. doi: 10.1111/jre.70098 (PMC13472043; doi:10.1111/jre.70098)
Supplement: Supplementary file 1 [file JRE-61-705-s001.docx]

**Supplementary figure S1.** **Representative macrophage gating strategy for flow cytometry (days 0, 3 and 6).** (A) Exclusion of debris, (B) selection of singlets, (C) determination of live cells (DAPI^-^), (D) M1 monocyte-derived macrophages (percentage HLA-DR^+^CD197^+^ cells) and (E) M2 monocyte-derived macrophages (percentage CD163^+^CD206^+^ cells).

**Supplementary figure S2. Cell viability during the polarization time course.** (A) Percentage de macrophages at baseline (D0) and under M0 conditions**.** (B) Percentage de macrophages at baseline (D0) and under M1 conditions**.** (C) Percentage de macrophages at baseline (D0) and under M2 conditions**.** Each box shows median values; box heights symbolize the IQR (Q3–Q1); Q3 and Q1 quartiles correspond to the top and bottom boundaries of the box, respectively; whiskers indicate values > Q3 (top: max - Q3) or < Q1 (bottom: Q1 - min). Intragroup differences were estimated using non-parametric Friedman's test and Conover's post test; intergroup differences used non-parametric Kruskal-Wallis, followed by Dunn's *post hoc*. Significant intragroup differences: * indicates *P < 0.05* compared with D0*;* Black bars show intergroup differences *(P < 0.05).* D = day; H = periodontal health; IQR = interquartile range; PIII-B = treated stage III, grade B periodontitis; PIII-C = treated stage III, grade C periodontitis.
